# Note Relative to the Existence of a Sexual Generation of Infusoria

**Published:** 1858-10

**Authors:** E. G. Balbiani

**Affiliations:** Member of the Society of Biology


### BUFFALO MEDICAL JOURNAL

AND

### MONTHLY REVIEW.

**VOL. 14.** OCTOBER, 1858. **NO. 5.**

### ORIGINAL COMMUNICATIONS.

ART. I.—*Note relative to the Existence of a Sexual Generation in Infusoria.* By Dr. E. G. BALBIANI, Member of the Society of Biology. Presented to the Academy of Sciences, Paris, March 29th, 1858. Translated from the "Journal de la Physiologie de l'homme et des Animaux," by the EDITOR.

The discovery of the propagation of infusoria by the production of embryons or internal germs, already established in a certain number of varieties belonging to several different groups, has opened a new field for research in the history of the development of these animalcules. It has shown, in fact, that besides the two modes of reproduction plainly agamous, by spontaneous division and by gemmation, till then alone admitted into that class, there existed a third, susceptible of an entirely different interpretation, and which had at least this point of resemblance with the reproduction by embryons of the higher sexual species, that in the same manner as these last, the young are formed in the interior, if not in a special cavity, of the parent which gives them birth. But no one, up to the present time, has yet shown that the formation of embryos, in the infusoria, was accompanied by any of the circumstances which characterize, in an indubitable manner, generation accomplished by the aid of distinct sexual organs. Stein, one of the first, has called attention to the part which the nucleus plays in this production; but he thought that the germs developed themselves on the surface of this body, by a process of budding, which made them resemble more the caducous buds or elevations, than embryos coming from fecundated eggs.

My personal observations have led me to view the origin of these bodies in a different light; I hope to be fortunate enough to demonstrate that the phenomena which accompany their formation, bring them completely in the order of those which essentially characterize sexual generation in the higher animals. Not being able to enlarge much in this "Note" on the facts which it has happened to me to observe, and which relate to six or seven species representing different groups, I will content myself with giving here a rapid sketch of the phenomena relative to the production by embryos of that one among them, where I have been able to follow them most completely, the green Paramecium, Paramecium bursaria of Focke *(Lexodes bursaria, Ehrb.)*

As in almost all the infusoria, there exists in this species a nucleus which is accompanied by a small lenticular body, ordinarily situated in an excavation of the nucleus near one of its extremities, and described generally under the improper name of nucleolus.

For several generations, the Paramecia multiply by spontaneous segmentation, each of the two new individuals obtaining a half of the primitive nucleus. Such is the very simple process of this mode of reproduction; but under the influence of conditions yet unknown, the species propagate in an entirely different manner, accompanied by phenomena much more complex than those which were apparent in multiplication by fissuring. In this new mode, we will see indicated, indeed, the real anatomical signification of the nucleus and nucleolus; the office of which, if we except the breaking of the first of these two organs in the act of the spontaneous division, has been till this time, entirely passive. It is, in fact, from them that the male and female reproductive elements, which characterize this mode of reproduction, go to form themselves.

When the time has arrived when the Paramecia must propagate by a concourse of the sexes, we see them collecting in certain parts of the vessel, either near the bottom or in the sides. The copulation is always preceded by certain preliminaries, quite curious to observe, but of which we cannot treat in this article. Very soon we see them coupled two by two, joined laterally, and as it were enlaced, their corresponding extremities directed in the same way, and the two mouths closely applied the one to the other. In this state, the two individuals joined together continue to move actively in the liquid, turning themselves constantly around their axis. Nothing announces, before copulation, the great changes which come to pass in the nucleus, and in the nucleolus which accompanies it. It is during this same copulation, the duration of which is prolonged for from five to six days and more, that their transformation is effected into sexual reproductive organs.

The nucleolus has undergone a considerable enlargement, and is transformed into a kind of capsule of an oval form, the surface of which is marked by longitudinal and parallel lines or striæ. Almost always, it divides without delay, following its long axis, into two, or more frequently, into four parts, which continue to grow independently of each other and in a very irregular manner, and constitute as many secondary pouches or capsules. At a time yet near this division, these last (capsules) show that they are composed of an extremely delicate membrane enveloping a bundle of small curved lines extending from one extremity of the pouch to the other, swelling near their middle and much diminished in size at the extremities. It is these which, seen through the enveloping membrane, give to the capsule the striated appearance which characterizes it, and which already exists in the nucleolus at almost all the other epochs of the life of the infusorium. It encloses, in addition, a perfectly colorless and homogenous liquid. What has become, during this time, of the nucleus? This has also changed its form and appearance; it has become rounded, enlarged; its substance, now softer, has lost its refracting quality, and presents, near its borders, curved fissures, which, penetrating deeper and deeper into its substance, separate one or more fragments in which there is an opening sufficient to show a certain number of small transparent spheres with a central dark point. At other times almost the entire nucleus presents this aspect, and then appears, as it were, stuffed with these small bodies, which are, without the least doubt, analagous to ovules. The evolution of the nucleus and nucleolus being identical, taking the same course in the two copulating infusoria, it results, (considering at this moment, the first as an ovary and the second as a testicle or seminal capsule) that not only each of them possesses the attributes of both sexes, but that they fecundate each other and act at one and the same time the part of male and female. As to this fecundation itself, every thing appears to show that it operates by means of an exchange which is made by the two individuals, of one or more of their seminal capsules, which pass from the interior of one of the infusoria to the other, through the closely applied orifices of the mouths; for we are often able, if not to see the very passage, at least to find a moment when one of the capsules, already engaged in one of the mouths, is on the point of going through the opening. Does the exchange from which the fecundation results, take place for all the capsules at one and the same copulation, in as many successive copulations with different individuals? We have here a question, the solution of which is not easy, and which, toconfine ourselves to the field of our observation, we will not here seek to solve.

Whatever it may be, each capsule, after its transmission, continues to enlarge in the interior of the individual which has received it, for we have never found any which had attained the extent of its development in infusoria yet coupled together. They then often attain a greater volume than the nucleus itself, but never arrive at maturity more than one at a time. When we examine one arrived at that state, after having pressed it from the body of the infusorium to disengage from it granules which always more or less obscure it, it appears in the form of a large ovoid-body, the surface of which presents a multitude of parallel striæ directed in the long diameter, and which result from a series of rows of corpuscles in its interior. Compression, pushed to the point of rupture, plainly shows it to be formed of a membrane of extreme tenuity, and the enclosed contents, an immense quantity of small fusiform corpuscles, the extremities of which are lost completely from our view by reason of their exceeding fineness. As soon as they are free, these little bodies are animated by a vibrating and propelling movement which leads very soon to their dispersion in the surrounding liquid. Evidently, we have here, the spermatozoids of the *Paramecium bursaria.* Iodine, alcohol, acetic acid, stop these movements instantaneously; they are insoluble in the last reagent in its concentrated form, which on the contrary, rapidly dissolves all the other elements of the body, excepting some green granules.

It is ordinarily from the fifth to the sixth day which follows copulation, that we see the first germs appear, in the form of small rounded bodies, composed of a membrane which is well brought to view by acetic acid, and greenish contents, pale, homogenous or almost imperceptibly granular, in which we can yet distinguish neither a nucleus nor contractile vesicle. It is not until later that these organs appear. The observations of Stein and of F. Cohn, have shown how the embryons left the body of the mother in the form of particles furnished with button shaped processes, real suckers, by means of which they remained adherent to her for some time, nourishing themselves from her substance; but these researches have not demonstrated the final separation of the young. I have been able to follow them sufficiently long after their detachment from the maternal body, and have been able to convince myself, that, after having lost their suckers, which are transformed into vibratile cilia, and a mouth being developed, which commences to show itself in the form of a longitudinal furrow, they finally came to the same form as the mother, containing the characteristic green granules of the Paramecia, without having undergone any more complicated metamorphoses.

NOTE.—We made the preceding translation from the excellent Physiological Journal of Brown-Séquard, in the hope that this new development in the physiology of generation would be of interest to all our readers. The study of generation is one of the most interesting, if not the most interesting department of physiology, and one which has excited the attention of the greatest men that have ever lived in the profession. The opinion has been that infusoria were developed originally by spores, floating about in the atmosphere in the form of a fine dust, which became developed into these little creatures, whenever they found a suitable receptacle. This is undoubtedly the fact, and it is also well known that infusoria multiplied themselves by spontaneous division, or what is called fissiparous generation, and gemmation or budding. It happens to every one who is in the habit of using a microscope, to see infusoria partially divided, especially the Paramecia, which are exceedingly common. The researches of Balbiani were made in this variety, which are of the first order of infusoria. His observations seem to have established an absolute sexual concourse, and he even states that he has discovered the spermatozoids. We know that in agriculture, vegetables which are propagated by gemmation, require a renewal of the original stock, or they will run out; and it has always seemed probable that the infusoria would likewise require a generation by ovulation from time to time, though they might most frequently multiply by spontaneous division or budding. These researches open a new field for investigation, which we hope will be occupied, and the observations carried to other classes of infusoria.—*Editor Buff. Med. Journal.*

